# Light availability impacts structure and function of phototrophic stream biofilms across domains and trophic levels

**DOI:** 10.1111/mec.14696

**Published:** 2018-06-04

**Authors:** Mia M. Bengtsson, Karoline Wagner, Clarissa Schwab, Tim Urich, Tom J. Battin

**Affiliations:** ^1^ Institute of Microbiology University of Greifswald Greifswald Germany; ^2^ Department of Limnology and Oceanography University of Vienna Vienna Austria; ^3^ Department of Health Sciences and Technology ETH Zürich Zürich Switzerland; ^4^ Stream Biofilm and Ecosystem Research Laboratory Ecole Polytechnique Fédérale de Lausanne (EPFL) ENAC Lausanne Switzerland

**Keywords:** diatoms, freshwater, metatranscriptomics, microbial eukaryotes, mRNA, rRNA

## Abstract

Phototrophic biofilms are ubiquitous in freshwater and marine environments where they are critical for biogeochemical cycling, food webs and in industrial applications. In streams, phototrophic biofilms dominate benthic microbial life and harbour an immense prokaryotic and eukaryotic microbial biodiversity with biotic interactions across domains and trophic levels. Here, we examine how community structure and function of these biofilms respond to varying light availability, as the crucial energy source for phototrophic biofilms. Using metatranscriptomics, we found that under light limitation‐dominant phototrophs, including diatoms and cyanobacteria, displayed a remarkable plasticity in their photosynthetic machinery manifested as higher abundance of messenger RNAs (mRNAs) involved in photosynthesis and chloroplast ribosomal RNA. Under higher light availability, bacterial mRNAs involved in phosphorus metabolism, mainly from Betaproteobacteria and Cyanobacteria, increased, likely compensating for nutrient depletion in thick biofilms with high biomass. Consumers, including diverse ciliates, displayed community shifts indicating preferential grazing on algae instead of bacteria under higher light. For the first time, we show that the functional integrity of stream biofilms under variable light availability is maintained by structure–function adaptations on several trophic levels. Our findings shed new light on complex biofilms, or “microbial jungles”, where in analogy to forests, diverse and multitrophic level communities lend stability to ecosystem functioning. This multitrophic level perspective, coupling metatranscriptomics to process measurements, could advance understanding of microbial‐driven ecosystems beyond biofilms, including planktonic and soil environments.

## Abstract 2

https://doi.org/10.1111/mec.14733

## 1 INTRODUCTION

Taxonomic and phylogenetic diversity in natural microbial communities is massive (Locey & Lennon, 2016; Torsvik, Goksøyr, & Daae, 1990). Despite the methodological leaps that have enabled description of this biodiversity, from deep sequencing of the prokaryotic rare biosphere (Sogin et al., 2006) to phylogenetic inference of novel prokaryotic and eukaryotic lineages (Hug et al., 2016; Jones et al., 2011), a tendency to address prokaryotes and eukaryotes separately has prevailed in most biodiversity studies. This inhibits the understanding of the ecology of complex microbial communities that include organisms across all domains of life on several trophic levels. Metatranscriptomics has the potential to overcome these barriers by addressing structure and function of entire microbial consortia while avoiding common biases associated with PCR amplification of marker genes (Urich et al., 2008). This is made possible by parallel analysis of small subunit ribosomal RNA (SSU rRNA), as a common currency for living biomass of all three domains of life and of the mRNA from actively transcribed functional genes providing insights into the functional capacity of the community. In addition, metatranscriptomics and related molecular techniques have advantages over traditional microscopic identification of (eukaryotic) morphospecies, which may underestimate microbial diversity due to time‐consuming manual counting of cells, and preferential detection of taxa with distinct morphologies over inconspicuous cells which can comprise hidden phylogenetic and functional diversity (e.g., Jones et al., 2011; Liu et al., 2009).

In complex microbial habitats such as soils, metatranscriptomics has contributed to the discovery of relatively abundant but previously largely undetected taxa (Geisen et al., 2015). Metatranscriptomics has also elucidated the functional adaptations of microbial communities in marine plankton, for example in response to iron limitation (Marchetti et al., 2012), and has helped to unravel complex interactions between diatoms and bacteria (Amin et al., 2015) and resource partitioning between diatoms (Alexander, Jenkins, Rynearson, & Dyhrman, 2015). For biofilms, metatranscriptomics has mainly been employed to study clinically relevant biofilms, such as gene expression in oral biofilms associated with periodontitis (Frias‐Lopez & Duran‐Pinedo, 2012; Yost, Duran‐Pinedo, Teles, Krishnan, & Frias‐Lopez, 2015). However, studies that link structure and function in complex environmental biofilms are rare (but see Lindemann et al., 2017), despite the importance of such biofilms for primary production, carbon and nutrient cycling in various aquatic ecosystems.

Stream biofilms are a prime example of phylogenetically diverse, multitrophic level communities. They form the trophic basis of the stream ecosystem contributing the bulk of primary production and biomass (reviewed in (Battin, Besemer, Bengtsson, Romani, & Packmann, 2016). Stream biofilms harbour diverse phototrophic algae, cyanobacteria, heterotrophic bacteria and various eukaryotic consumers, among them even early‐instar insect larvae. Several studies have highlighted the phylogenetic diversity and important roles of each of these functional groups separately (Besemer et al., 2013; Bott & Kaplan, 1990; Schmid‐Araya, 1994; Wellnitz & Rader, 2003), yet only rarely in combination (Piggott, Salis, Lear, Townsend, & Matthaei, 2014; Romani et al., 2014). Thus, there is limited understanding of how environmental variability and change affects structure and function of organisms of different biofilm functional groups simultaneously, including interactions between them, and how this influences their collective contribution to biogeochemical fluxes.

Light is a primary resource in streams, which fuels photosynthesis by algae and cyanobacteria in biofilms and therefore lays the basis of the carbon (C) and nutrient (N and P) cycles in streams in addition to terrestrial C inputs (allochthonous C). Although the importance of light in regulating stream food webs and biogeochemical fluxes is widely acknowledged, most studies to date have focused on one or a few functional groups when assessing the effects of altered light regimes on stream organisms (Hill, Ryon, & Schilling, 1995; Romani et al., 2014; Ylla, Borrego, Romani, & Sabater, 2009). For example, several studies have demonstrated that stream biofilm phototrophs (also referred to as periphyton) are colimited by light and phosphorus (P; Hill & Fanta, 2008; Hill, Roberts, Francoeur, & Fanta, 2011). According to the light:nutrient hypothesis (Sterner, Elser, & Fee, 1997), alterations in these resources affect periphyton C:P ratios, with implications for higher trophic levels and ecosystem function (Fanta, Hill, Smith, & Roberts, 2010; Hill, Rinchard, & Czesny, 2011). As predicted by this hypothesis, increased production and exudation of C‐rich photosynthates by phototrophs under high light, P‐depleted conditions, also fuel bacterial heterotrophic metabolism. However, it is currently unclear how this affects the uptake of limiting P by phototrophs and heterotrophic bacteria in the biofilm. Moreover, increased light availability may cause a shift in the relative importance of autochthonous (stream phototroph‐derived) and allochthonous (terrestrial‐derived) carbon sources for bacterial heterotrophs, with unknown consequences for biofilm structure and function. Understanding how light modulates stream biofilm structure and function is fundamental, as global climate change and human activities alter light regimes in many streams through for example deforestation (increased light), but also through upward shifts of the tree line in alpine areas due to global warming (decreased light; Lenoir, Gegout, Marquet, de Ruffray, & Brisse, 2008). Ultimately, light availability in stream biofilms is analogous to primary resource variability in many microbially driven ecosystems. For example, planktonic communities experience resource variability in light and nutrient levels resulting in bloom and nonbloom states with significant impacts on structure and function of both algal, bacterial and consumer communities. In soils, variations in above‐ground primary productivity also affect microbial biomass and community composition, and vice versa (Wardle et al., 2004). Despite this, studies investigating the impacts of resource variability on several microbial functional groups and trophic levels at the same time remain rare in any ecosystem, hindering insights into the links between structure, function and biogeochemical processes in complex microbial communities.

We experimented with phototrophic biofilms initially grown in headwater streams and further incubated in microcosms to determine how light availability impacts structure and function of biofilms across all three domains of life, assessed using metatranscriptomics. We expected that light availability would impact all microbial functional groups, leading to shifts in both community composition and function. More specifically, we hypothesized that functional groups (i.e., phototrophs, heterotrophic bacteria and microbial consumers) would respond differently reflecting their unique roles within the biofilm matrix:

- Overall, we expected that shifts in community composition in response to light availability would be more pronounced for phototrophs than for heterotrophic bacteria and consumers, as the latter functional groups should display only a secondary response to light mediated by changes in phototroph biomass, activity and community composition.
- Phototrophs (algae and cyanobacteria) should display a shift in community composition to more shade‐tolerant taxa under lower light availability accompanied by functional shifts in expression of genes involved in photosynthesis. Additionally, genes involved in P uptake should be overexpressed in treatments with higher light availability, reflecting the lower availability of this limiting nutrient relative to light (Sterner et al., 1997).
- Heterotrophic bacteria should also display a shift in community composition due to increased availability of photosynthetic exudates in higher light availability relative to allochthonous C sources. Moreover, genes involved in P uptake should be overexpressed in higher light availability as a result of reduced relative P availability and increased bacterial P demand due to metabolic stimulation by increased phototroph exudation.
- Biofilm consumers such as microbial grazers will experience a shift in their food source as light availability alters the community composition within the phototroph and heterotrophic bacteria functional groups, causing a secondary shift in the community composition of consumers.

## 2 MATERIALS AND METHODS

### 2.1 Biofilm establishment

Biofilms were grown in a flume with diverted stream water from the Oberer Seebach (Lunz am See, Austria) during a period of 4 weeks in June 2012. Glass tiles of approximately 1 cm^2^ were used as a substrate and were covered by shading foils to manipulate the light availability for the growing biofilms. Biofilm‐covered tiles from three different light conditions, 92%, 51% and 7% light transmission, were transferred into triplicate microcosms. For a detailed explanation of the biofilm establishment, see Wagner, Besemer, Burns, Battin, and Bengtsson (2015).

### 2.2 Microcosm incubations

Using laboratory microcosm incubations after biofilm establishment, we could measure the contribution of the biofilms to essential processes such as primary production and nutrient uptake. Microcosms consisted of 0.74 L, gas‐tight transparent plexiglas chambers, equipped with magnetic stirring and a perforated support which accommodated the biofilm‐covered glass tiles (Wagner et al., 2015). The top of each microcosm was covered with the respective shading foil (92%, 51% and 7% light transmission), and all microcosms were illuminated with the same fluorescent lights. The resulting light intensities ranged between 10 and 150 micromole photons m^−2^ s^−2^ with slight variability within the three light treatments caused by differences in the placement of microcosms in relation to the light source (Table 1). The light intensities are within the variability that may be encountered in a stream draining a forested catchment, where riparian vegetation shades the streambed from direct sunlight (Hill et al., 1995). Microcosm incubations were carried out in a climate chamber at 17.5°C (±2°C). There was no difference in temperature between light treatments, yet the temperature was elevated in all treatments compared to stream conditions during biofilm establishment (10.2°C ± 1.6°C). Each microcosm was filled with 90 tiles (each ~1 cm^2^) at the start of the 7‐day experiment, and three tiles were harvested on days 1, 3 and 7, flash‐frozen in liquid N_2_ and stored at −80°C until nucleic acid extraction. Microcosms were filled with a stream water medium consisting of raw oligotrophic groundwater from the region supplemented with an aged extract of crack willow (*Salix fragilis*) leaves to mimic terrigenous DOM (Wagner et al., 2014). The entire volume in each of the microcosms was exchanged at the start of each day and night incubation, which lasted for 6 hours. Concentrations of O_2_, DOC, NO_3_ and PO_4_ were measured at the start and end of each day and night incubation (as described in Wagner et al., 2015), and the uptake/release of solutes was calculated as the difference between start and end, normalized to the biofilm area and expressed as an hourly rate. Gross primary production was calculated from the daytime oxygen release (net primary production) compensating for the oxygen uptake during the following night (respiration). Biofilm biomass and C content were determined on 6 cm^2^ of biofilm from each treatment (freeze‐dried) at the end of the incubations using an Elemental Analyzer (EA1110, CE Instruments, Thermo Fisher).

**Table 1:** Biofilm biomass parameters and biofilm‐mediated fluxes in response to light availability. Values represent means of treatment replicates (*n* = 3), ±1 *SD*. All light treatments represent light levels typical of stream reaches shaded to various degrees (not exposed to full sunlight)

Treatment	Light intensity (μmol photons s^−1^ m^−2^)	Bacterial cells (cells/cm^2^)	Chlorophyll *a* (μg/cm)	Biomass dry weight (mg/cm^2^)	Gross primary production (mg C cm^−2^ h^−1^)	PO_4_ uptake (μg P(PO_4_) cm^−2^ h^−1^)
High light	125.4 ± 29.1	1.1e+8 ± 4.8e+6	4.3 ± 0.92	4.8 ± 0.06	6.13 ± 0.69	0.12 ± 0.01
Intermediate light	32.9 ± 9.1	7.8e+7 ± 1.9e+7	4.2 ± 0.5	3.0 ± 0.65	4.40 ± 0.81	0.10 ± 0.003
Low light	11.1 ± 1.8	3.0e+7 ± 1.1e+6	2.1 ± 0.14	1.6 ± 0.21	1.85 ± 0.62	0.05 ± 0.01

### 2.3 Metatranscriptomics

Total nucleic acids (NA) were extracted from each tile with a phenol–chloroform‐based method featuring mechanical lysis (Urich et al., 2008). The extracts of tiles from the different time points from each microcosm were pooled using equal NA amounts. Thus, one time‐integrated sample from each microcosm (*n* = 9) comprised NA extracted from approximately 9 cm^2^ of biofilm. Nucleic acid extracts were treated with DNAse, and RNA was purified using the AllPrep kit (Qiagen) and subjected to linear amplification using MessageAmp II kit (Ambion, Tveit, Urich, & Svenning, 2014). Double‐stranded cDNA was prepared using the SuperScript Double‐Stranded cDNA Synthesis Kit (Invitrogen). Barcoded, adapter‐ligated cDNA libraries were generated and were sequenced by a PGM IonTorrent (Life Sciences) using 200 bp sequencing chemistry and 318 chips according to instructions supplied by the manufacturer. Sequence reads were size‐selected (>100 bp) before further processing. Sequencing yielded between 830 341 and 1 594 422 reads for each library.

### 2.4 Bioinformatics and statistics

Metatranscriptomic reads were sorted into ribosomal RNA and putative mRNA using sortmeRNA (Kopylova, Noé, & Touzet, 2012). Small subunit rRNA reads (SSU rRNA) were aligned against the CREST Silvamod reference database (Lanzén et al., 2012) using BLASTN (Altschul, Gish, Miller, Myers, & Lipman, 1990) and were assigned taxonomically using MEGAN (Huson, Auch, Qi, & Schuster, 2007) (LCA parameters: Min Score 100, Max expected 0.01, Top per cent 5, min support 1, LCA per cent 100) using a per cent identity filter for 16S rRNA. Putative mRNAs were functionally annotated according to SEED Subsystems and taxonomically assigned with best‐hit classification using the MGRAST online pipeline (Meyer et al., 2008).

Ribosomal RNA reads classified on different taxonomical levels were exported from MEGAN, while function‐assigned reads (mRNA) were exported from MGRAST. To link functional gene tags to microbial taxa, functionally annotated and taxonomically assigned mRNA reads were exported from MGRAST using the workbench feature. To address differential abundance of microbial taxa (rRNA) and differential expression of microbial functional categories (mRNA) in response to light, read count data were analysed using a statistical framework for RNA‐Seq data implemented in the edger package in r (R Development Core Team, 2018; Robinson, McCarthy, & Smyth, 2010). Specifically, tag‐wise dispersion was estimated using negative binomial generalized linear models. Differential expression between light treatments was determined using pairwise likelihood ratio tests correcting for multiple testing with the Benjamini–Hochberg method. For all analyses of microbial taxa, reads originating from Chironomid larvae (classified as *Insecta*) were removed from the data set to reduce the influence of their stochastic distribution among samples owing to the macroscopic size of these organisms. To visualize the overall distribution of taxa (rRNA, including Chironomids) and functional gene categories (mRNA) in the entire data set, we used hierarchical pie charts generated by Krona (Ondov, Bergman, & Phillippy, 2011).

Taxa (genus level) were classified as dominant (top 50 most abundant taxa) and subdominant (top 51st to 100th most abundant taxa) according to their overall relative abundance across all nine samples. Distance‐based RDA (Bray–Curtis distance) ordination (function capscale) was used to display variability in dominant and subdominant taxa as well as mRNA functional categories (SEED subsystems level 2). The variation explained by light was assessed using PERMANOVA (function adonis). Procrustes analysis was used to detect correlations between taxa‐ and mRNA variability (functions procrustes and protest). The r functions are contained in the vegan package (Jari Oksanen et al., 2018).

## 3 RESULTS AND DISCUSSION

### 3.1 Light availability influenced biofilm‐mediated biogeochemical fluxes

**Figure 1:**
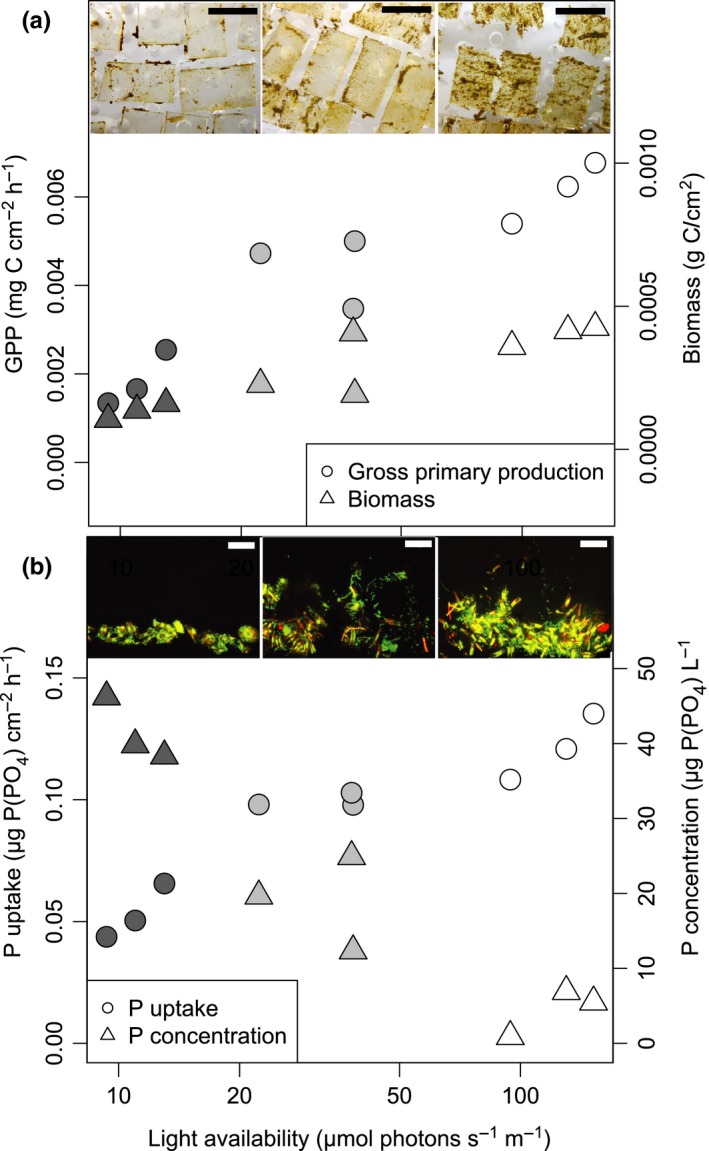
The effect of light availability on biofilm‐mediated fluxes and physical appearance. (a) Increased light availability led to a threefold increase in gross primary production (GPP) and biofilm biomass, also visible macroscopically as thicker biofilms (inset, scale bar 1 cm). (b) Daytime uptake of phosphate (P‐PO_4_) was elevated in biofilms grown under higher light availability, and these biofilms also depleted the phosphate concentration inside the microcosms during the 6‐hr incubations. For GPP, P‐PO4 uptake and concentration, every point represents a mean of several individual measurements during the week‐long experiment from one individual microcosm. Biomass was measured at the end of the experiment. Shading of the plotting points indicates light treatment (darker shade = lower light intensity). The inset shows vertical cross sections of biofilms with algal and bacterial cells captured by confocal laser scanning microscopy. Green colour represents nucleic acid stain SYTOX green (bacterial cells and eukaryotic nuclei), while red colour represents chlorophyll *a* autofluorescence. Scale bar: 20 μm [Colour figure can be viewed at http://wileyonlinelibrary.com]

We found that experimental shading resulted in several changes in biofilm properties and biofilm‐mediated fluxes, summarized in Table 1. Notably, primary production and biofilm biomass were reduced by a factor of 3 between the highest and the lowest light availability treatments, also visible to the naked eye as a thicker biofilm cover in the highest light availability treatment (Figure 1a). Likewise, phosphate (P‐PO_4_) uptake was strongly affected by light intensity (Figure 1b). However, even under very low light intensity (<10 micromole photons m^−2^ s^−2^), biofilms remained net phototrophic and removed significant amounts of P‐PO_4_ during microcosm incubations (Figure 1a,b). This indicates that these biofilms are well adapted to low light availability, allowing them to persist also under shaded conditions which occur for example in forested catchments and on the underside of larger boulders and rocks in the streambed.

### 3.2 Shifts in community composition were especially pronounced for subdominant biofilm taxa and were linked to shifts in functional gene expression

**Figure 2:**
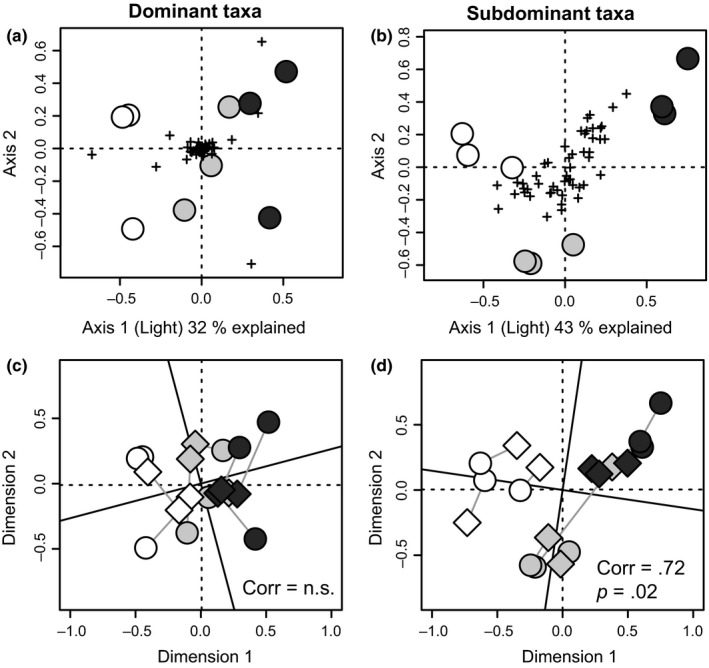
The response of dominant and subdominant taxa to varying light availability. The RDA ordinations display variability in sample composition of dominant (a,c) and subdominant (b,d) genus‐level taxa (based on rRNA abundance). Ordinations were constrained to light (measured light intensity), which varies along the *x*‐axis. Circles represent samples, and crosses represent taxa (a–b). In c–d, the taxa‐based ordinations (circles) have been correlated to an ordination of functional mRNA categories (SEED Subsystems level 2) of the same samples (diamonds) using Procrustes rotation. Grey lines connect taxa (rRNA)‐based and mRNA‐based points from the same sample. Correlation statistics are indicated in the plots. Shading of the plotting points indicates light treatment (darker shade = lower light intensity). Dominant taxa are the top 50 most abundant genus‐level taxa in the data set, while subdominant taxa were defined as the 51st to 100th most abundant taxa

Metatranscriptomic analysis of SSU rRNA and mRNA revealed a clear shift in response to light availability for genus‐level taxa of all functional groups (Figure 2a,b) and for biofilm function (Figure 2c,d), consistent with our initial hypothesis. As expected, light availability caused a more pronounced community composition shift among phototrophs (PERMANOVA *R* ^2^= 0.43, i.e., 43% of community variation explained, *p* = .02) than among heterotrophic bacteria (30% of variation explained, *p* = .02) and consumers (35% of variation explained, *p* = .02). Interestingly, light was a better predictor for whole community variation (43% of variation explained, *p* = .01) for subdominant taxa (Figure 2b, defined as the 51st to 100th most abundant taxa, Table S1) than for dominant taxa (Figure 2a, defined as top 50 taxa, 32% explained, *p* = .01, Table S1). This implies that the relative abundances of dominant biofilm taxa remained rather stable across the light gradient, while shifts among subdominant taxa were more pronounced. Biofilm function, assessed by mRNA transcripts, also varied with light, which explained 22% (*p* = .02) of variation in mRNA functional categories (SEED Subsystems level 2). Variation in structure (rRNA) and function (mRNA) was correlated for subdominant taxa (*p* = .02, Figure 2d), but not for dominant taxa (Figure 2c), which illustrates the link between structure and function in these biofilms. However, variability in functional gene expression does not automatically reflect the contribution of taxa to overall biofilm function, measured as biogeochemical fluxes, as dominant taxa may regulate their functional gene expression in response to light availability without corresponding changes in abundance.

### 3.3 Biofilms were dominated by diatoms and cyanobacteria

**Figure 3:**
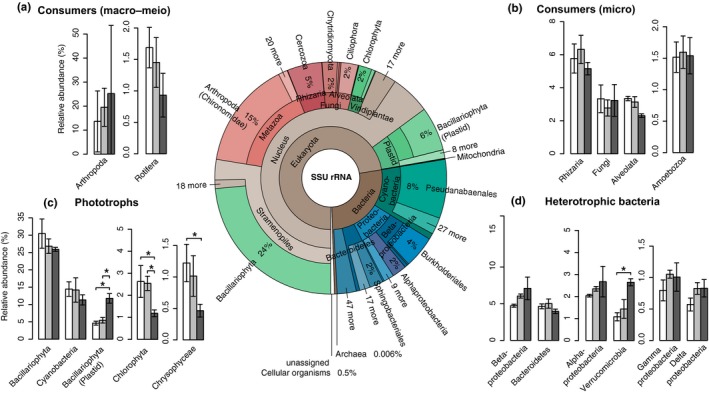
The relative abundances of the main biofilm taxa on a broad taxonomic level. The Krona chart (centre) displays overall relative abundance of SSU rRNA‐derived sequencing reads of all samples (*n* = 9). Taxa are coloured according to functional group: Phototrophs = greens, consumers = reds and heterotrophic bacteria = blues (brown shades indicate higher‐level taxa encompassing several functional groups). The bar plots (a–d) display relative abundances of specific taxa in response to the different light conditions (normalized to assigned SSU reads per sample and expressed as a percentage). Taxa are displayed according to major functional groups: Consumers (a: macro–meio consumers, b: microconsumers), phototrophic taxa (c) and heterotrophic bacteria (d). Bars are shaded to indicate the light condition (darker shade = lower light intensity). Error bars refer to standard deviation of the mean (*n* = 3), and significant differences between the light treatments are indicated with asterisks (*p < .05) according to taxa‐wise negative binomial GLMs with Benjamini–Hochberg correction for multiple testing (as implemented in edger)

**Figure 4:**
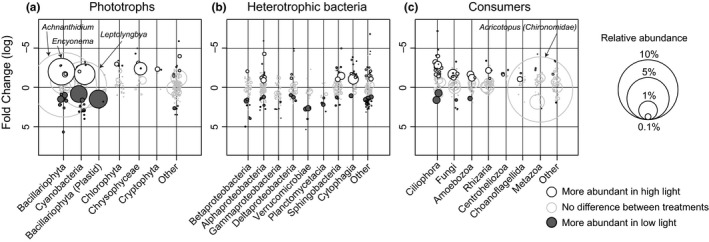
The response of genus‐level taxa of the biofilm functional groups phototrophs (a), heterotrophic bacteria (b) and consumers (c) to light availability. Every circle represents a genus‐level taxon, with the area of the circle scaled to the average relative abundance of the taxon across all light treatments. The position along the *y*‐axis shows the average fold change of taxa in the highest light vs. lowest light availability treatments. The taxa are grouped by major taxonomical groups (phylum–class level), except for low‐abundance taxa which are collected in the artificial group “other” for each functional group. White and shaded circles with black outline represent taxa that are significantly more abundant in the highest light and lowest light availability treatment, respectively. Circles with a grey outline represent taxa that do not differ significantly in abundance between the highest and lowest light availability treatments. Selected taxa that are mentioned in the text are pointed out with arrows

Dominant taxa were found among eukaryotes and bacteria in the biofilms, while archaea represented only a minor fraction (<0.01%) of the community (Figure 3 centre). Diatoms (*Bacillariophyta*) dominated the algal community and constituted on average 30%–35% of SSU rRNA including plastid SSU rRNA (Figure 3 centre, c). The most abundant genus was *Achnanthidium* (Figure S1, Figure 4a), which agrees with previous microscopic identification of the diatom *Achnanthidium minutissimum* as an abundant benthic species in the same stream that we grew the biofilm in (Adlboller, 2013). Diatoms are the major building blocks of these biofilms (Battin, Kaplan, Newbold, Cheng, & Hansen, 2003), contributing to the production of extracellular polymeric substances (EPS, Bahulikar & Kroth, 2007) and through their photosynthetic exudates providing labile resources for the bacterial heterotrophs that live in their close proximity. Furthermore, we detected abundant cyanobacterial rRNAs classified to the genus *Leptolyngbya* (Figure S2, Figure 4a) which, because of their filamentous morphology, may also be important for biofilm architecture, providing complexity to the biofilms which can contribute to supporting the high biodiversity encountered on a relatively small area (9 cm^2^ of biofilm).

### 3.4 Dominant phototrophs responded to light limitation through shifts in expression of functional genes involved in photosynthesis

**Figure 5:**
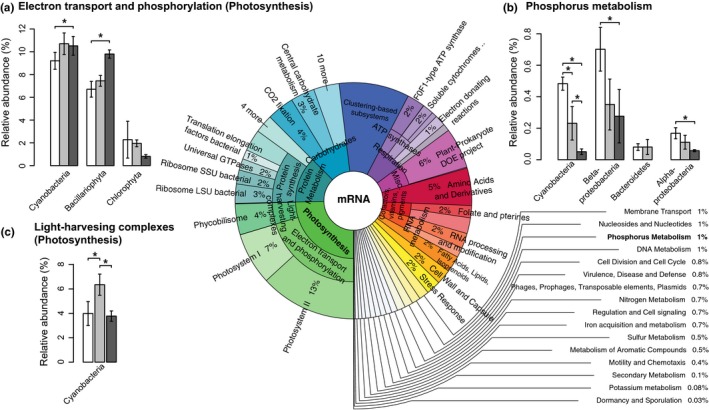
The relative abundances of biofilm functional gene transcripts. The Krona chart (centre) displays overall relative abundance of mRNA reads assigned to functional categories (SEED subsystems) of all samples (*n* = 9) while a–c displays mRNA reads involved in the key functions photosynthesis (a,c) and phosphorous metabolism (b) in response to the different light conditions. Read counts were normalized to assigned mRNA reads per sample and expressed as a percentage. Bars are shaded to indicate the light condition (darker shade = lower light intensity). Error bars refer to standard deviation of the mean (*n* = 3), and significant differences between the light treatments are indicated with asterisks (*p < .05) according to subsystem‐wise negative binomial GLMs with Benjamini–Hochberg correction for multiple testing (as implemented in edger)

Contrary to our expectations, the dominant phototroph taxa did not vary significantly in response to light availability (Figure 3c, Figure 4a). However, as we hypothesized, there was a shift in the expression of functional genes involved in photosynthesis (Figure 5a,c). For diatoms, these were genes involved in electron transport and phosphorylation which were significantly upregulated in the lowest versus the highest light availability treatment (0.5‐fold change, *p* = .05). This upregulation of genes involved in photosynthesis was paralleled by an over twofold increase (*p* < .001) in diatom chloroplast‐encoded 16S rRNA (Figure 3c), suggesting that the dominant diatoms increased their chloroplast translational activity to compensate for light limitation (Depauw, Rogato, Ribera d'Alcala, & Falciatore, 2012). Similarly, cyanobacterial mRNAs involved in electron transport and phosphorylation (Figure 5a) as well as synthesis of light‐harvesting complexes also displayed significant differential expression in response to light availability, the latter peaking at intermediate light intensities (Figure 5c). This suggests that the dominant diatoms and cyanobacteria possess high phenotypic plasticity with regards to their photosynthetic machineries (Figure 5a,c) which may allow them to not only persist, but to dominate biofilms even at very low light intensities (Figure 3c) and carry out the primary production which is essential for supporting other trophic levels such as heterotrophic bacteria and eukaryotic consumers.

### 3.5 Heterotrophic bacteria were more abundant under low light availability

Although diatoms remained dominant throughout the light gradient (Figure 3c), the relative abundance of heterotrophic bacteria significantly increased (0.35 fold, *p* < .001) under low light availability compared to higher light availability (Figure S3, blue shades). This is consistent with an increase in heterotrophic processes such as uptake of allochthonous (i.e., nonalgal) organic matter and detrital decomposition, which gained in importance under reduced light as shown in a parallel study (Wagner, Bengtsson, Findlay, Battin, & Ulseth, 2017). Overall, the composition of the bacterial communities largely agrees with previous results obtained with PCR‐based methods as summarized in (Battin et al., 2016), identifying taxa within *Betaproteobacteria*,* Alphaproteobacteria* and *Bacteroidetes* as particularly abundant community members (Figure 3 centre, d). This overall composition remained relatively stable along the light gradient, yet was complemented by considerable shifts of genus‐level taxa (Figure 4c, Table S2). For example, several genus‐level taxa within *Betaproteobacteria* and *Verrucomicrobia* increased in abundance when light availability and hence primary productivity were low (Figure 4c, Table S2); they may be primarily involved in decomposition of allochthonous or detrital organic matter (Newton, Jones, Eiler, McMahon, & Bertilsson, 2011).

A parallel study employing the same microcosms demonstrated a temporal shift in the bacterial communities during the week‐long experiment (Wagner et al., 2015). Due to our time‐integrated sampling strategy (time points were pooled owing to the relatively time‐ and resource‐intense sample processing for metatransciptomics), we cannot address temporal shifts in structure and function that may be caused for example by natural succession or temperature and light regime differences between the stream and the microcosms. Our results therefore reflect changes in response to light availability that are strong enough to be detected despite possible temporal shifts.

### 3.6 Higher light availability led to increases in expression of functional genes involved in phosphorus metabolism in bacteria

Thick biofilms developing in our high light treatment depleted P‐PO_4_ concentrations in the experimental microcosms (Figure 1b), which likely also led to even more severe local phosphorous (P) depletion within the biofilm where mass transfer becomes the limiting step compared to thinner biofilms (Battin, Kaplan, Newbold, & Hendricks, 2003). As in many oligotrophic freshwater ecosystems, P is the limiting inorganic nutrient in our study stream, where it puts constraints on primary production and biomass build‐up. P limitation as a result of increased light availability is a key prediction of the nutrient‐light hypothesis (Sterner et al., 1997), and we hypothesized that P limitation would lead to changes in expression of functional genes involved in P uptake. Biofilms exposed to the highest light availability indeed displayed a 1.5‐fold increase (*p* < .05) in relative abundance of mRNAs related to P metabolism (Table S3). The protein functions contained within P metabolism were diverse but included periplasmic phosphate‐binding proteins (TC 3.A.1.7.1), PO_4_ transport system regulatory proteins and alkaline phosphatase (EC 3.1.2.1). Most of the mRNAs involved in P metabolism were classified as bacterial, including cyanobacterial (Figure 5b, Table S3) highlighting the important role of the smallest cells, with a high‐surface‐area‐to‐volume ratio, in nutrient uptake and recycling within the biofilm. With some exceptions, the most abundant bacterial groups in the biofilms (according to rRNA relative abundance) also contributed most to expression of mRNAs involved in phosphorus metabolism. This includes *Cyanobacteria*,* Betaproteobacteria* and *Alphaproteobacteria*, while *Bacteroidetes* and *Verrucomicrobia* who were also abundant, appeared to contribute less (Figure 5b). The increase in expression of mRNAs involved in phosphorous metabolism under higher light availability seems independent from shifts in bacterial community composition as rRNA relative abundance of *Betaproteobacteria* and *Alphaproteobacteria* in fact decreased (though not significantly) under higher light availability (Figure 3d), and *Cyanobacteria* (Figure 3c), primarily comprised of the genus *Leptolyngbya* (Figure 4a), remained unchanged. This indicates that the intensified bacterial P metabolism is a physiological response of the bacterial cells when they are faced with phosphorous limitation due to a higher P demand of thick biofilms grown under higher light availability. The lack of mRNAs involved in P metabolism from diatoms may indicate that they are less responsive to P limitation compared to their bacterial neighbours, possibly instead relying on recycling of P resources by bacteria. This could point towards a mutualistic interaction between diatoms and heterotrophic bacteria within the biofilms, where diatoms provide photosynthetic exudates to heterotrophic bacteria in exchange for recycled limiting nutrients. This type of interaction has been extensively explored in planktonic systems (e.g., Amin, Parker, & Armbrust, 2012; Bratbak & Thingstad, 1985) yet may be even more relevant in biofilms due to the close proximity of algal and bacterial cells allowing exchange of solutes within the biofilm matrix. However, we cannot exclude the possibility that mRNAs involved in P metabolism could not reliably be assigned to diatoms due to the low representation of annotated diatom genomes in the public databases compared to bacteria.

### 3.7 Community shifts in eukaryotic biofilm consumers may reflect shifts in abundance of their prey along the light gradient

We detected diverse eukaryotic micrograzers, including ciliates (*Ciliophora*), *Cercozoa* and *Amoebozoa*, and even larger predators such as chironomid larvae and rotifers (Figure 3 centre). In addition, we detected many potentially parasitic taxa, such as chytrid fungi (*Chytridiomycota*) and vampyrellid rhizarians, which likely reduce the productivity of the dominant algae (Kagami, Miki, & Takimoto, 2014). On average, these consumers comprised 15%–25% of the biofilm SSU rRNA (Figure 3 centre, Figure S3, red shades), and the relative abundance of this functional group as a whole did not vary along the light gradient, but appeared to be relatively stable compared to phototrophic eukaryotes and bacteria (Figure S3). However, as hypothesized, light availability induced striking community shifts within specific taxa. For example, among the ciliates, several genera increased or decreased significantly with light (Figure 4c, Figure S4), suggesting shifts in functional feeding traits that possibly reflect the higher abundance of bacterial prey under low light availability. Thus, top‐down control by eukaryotic micrograzers may promote a balance between autotrophic and heterotrophic biofilm inhabitants, thereby contributing to the stability of biofilm function and maintenance of diversity along the steep light gradient, similarly to what has been proposed for macroscopic food webs (e.g., McCann, 2000).

### 3.8 Biofilm functional groups responded to light availability in unique ways

As detailed above, all functional groups responded to light availability through community shifts, consistent with our hypotheses. However, surprisingly, the most abundant algal and cyanobacterial taxa did not change in relative abundance (rRNA) along the light gradient, although they are directly affected by light through photosynthesis. Instead, they responded phenotypically, reacting to light limitation through increased expression of photosynthetic functions and an increased translational activity in diatom chloroplasts. Several less abundant algal and cyanobacterial taxa did however change significantly with light availability, which indicates a certain niche separation of taxa along light gradients. In addition, we observed strong shifts in genus‐level taxa in both heterotrophic bacteria and eukaryotic consumers, which indicates that they are affected by light either indirectly through the changed physiology or composition of the phototrophs, or directly through harvesting of light as an energy source. Mixotrophy and photoheterotrophy are widespread among both bacteria and eukaryotes (Kolber et al., 2001; Stoecker, 1998) and may contribute to the observed patterns to some extent.

Studies that link structure and function of phototrophic biofilms on a molecular level are rare, yet the few existing studies report a composition of organisms and gene expression under stable conditions that is a broadly similar to our results (Krohn‐Molt et al., 2013; Nakamura et al., 2016; Schneider, Reimer, Hahlbrock, Arp, & Daniel, 2015). In a recent study, Lindemann and colleagues investigated protein expression patterns during succession in cyanobacterial biofilms (Lindemann et al., 2017). They found that P starvation during 28 days of succession caused expression of similar P uptake genes in all bacteria, while nitrogen (N) uptake strategies varied more among microbial taxa. They suggest that, under severe P limitation, niche complementarity allowing for coexistence may be achieved by differing N uptake strategies.

In contrast to the sparsity of biofilm “omics” studies, a considerable body of literature deals with plankton, including conditions characterized by contrasting resource availability such as phytoplankton blooms and diel dynamics. In a diatom‐dominated marine phytoplankton bloom, phosphorous depletion was followed by increased expression of several different bacterial phosphorous acquiring proteins, showing that bacteria are essential for P cycling under high resource (C) availability also in this environment (Teeling et al., 2012). In an oligotrophic mountain lake, bacteria appeared to employ different P acquiring strategies during day (high resource availability) than during night (low resource availability), relying on inorganic P during the day and organic P during the night (Vila‐Costa, Sharma, Moran, & Casamayor, 2012). Although we found no indications of a similar P uptake strategy in our high‐light vs. low‐light‐grown biofilms in this study, we did detect an intensified utilization of allochthonous carbon during the night and under darker conditions in a parallel study from the same experiment (Wagner et al., 2017). This indicates that scavenging of organic molecules during dark conditions may release additional P to biofilms.

### 3.9 Ecological implications of the functional integrity of biofilms along changing light regimes

In summary, we have shown that functional groups of microorganisms that span several trophic levels and phylogenetic domains in stream phototrophic biofilms are affected by the availability of light in unique ways, ranging from community shifts (especially evident in heterotrophic bacteria and eukaryotic consumers) to physiological adaptations in algae and bacteria. However, the overall composition of the biofilm, characterized by a dominance of diatoms and a high diversity of bacteria and consumers, remained similar across the light gradient while shifts in response to light intensity mainly took place among subdominant and low‐abundance taxa. Importantly, biofilms maintained their functional integrity despite low light availability, likely mainly due to phenotypic plasticity of dominant phototrophic taxa. This implies that, although changing light regimes in streams will impact total primary production and nutrient cycling, reflecting the change in energy input and resulting biomass build‐up, biofilms are functionally adapted to persist and carry out essential biogeochemical processes even under very limiting light. This resilience of biofilm communities likely contributes to the stability of stream ecosystems, and their ability to recover from events that alter light regimes on the short term (such as shading by suspended solids after storms), and adapt to long‐term changes, including effects of riparian de‐ and reforestation (Hill et al., 1995; Stephenson & Morin, 2009).

Variations in primary resource availability are ubiquitous in nature. As already mentioned, phytoplankton blooms result from seasonal changes in light and nutrient availability. In soils, variations in inputs from above‐ground primary production cause differing resource availability for soil microbial communities (Wardle et al., 2004). There are some notable and fundamental differences between these situations and the stream biofilms that we have experimented on under variable light conditions. For example, while we observed a stable relative contribution of dominant phototroph taxa along the light gradient, phytoplankton blooms are characterized by a distinct succession of dominant taxa as the bloom proceeds, likely due to the different temporal and physiochemical mechanisms underlying blooms in addition to just light availability. On the other hand, soils exposed to reduced organic resource input due to tree girdling experience a rapid drop in microbial biomass (Dannenmann et al., 2009), but not necessarily in microbial community composition (Dannenmann et al., 2009; Wu et al., 2011). It is possible that biofilm and soil microbial communities are more buffered against changes in primary resource input than planktonic communities, responding mainly with changes in biomass rather than community composition. If so, it may be due to their structured nature, where microbial cells coexist in close proximity, offering opportunities for interactions (Shade et al., 2012). In the case of the stream biofilms studied in this work, a few dominant phototrophic taxa with high phenotypic plasticity appear to confer this stability by providing a structured habitat and fixed carbon to numerous biofilm heterotrophs, possibly in exchange for P recycling by biofilm bacteria.

### 3.10 Biofilms as “microbial jungles”

Our view into the structure and function of complex biofilms across all domains of life, which was enabled by metatranscriptomics, highlights that structured microbial consortia in nature are best understood as multitrophic entities whose function is determined by microbial interactions across trophic levels and domains. Such “microbial jungles” are the rule rather than the exception in microbial habitats, which rarely exclusively contain organisms from one domain or functional group (such as algae) although it may seem convenient to limit the scope when studying these communities. Analogous to macroscopic jungles where trees provide the three‐dimensional structure that houses tremendous biodiversity (Ellison et al., 2005; Lowman & Rinker, 2004), dominant diatoms and cyanobacteria in stream biofilms act as foundation taxa that build a structured habitat facilitating other organisms such as heterotrophic bacteria and microscopic consumers. We have shown how these dominant phototrophs offer stability to the biofilm via their phenotypic plasticity, similarly to how long‐lived trees in a forest withstand short‐term climatic variability and thereby protect its associated flora and fauna (Bormann & Likens, 1994). Long‐term diversity and stability in forests can be governed by species interactions such as insect herbivory and fungal pathogenicity (Bagchi et al., 2014; Janzen, 1970), whereas in biofilms, microscopic consumers such as ciliates and fungal parasites may regulate biomass and diversity. In conclusion, phototrophic biofilms contain more diversity than just the dominant phototrophs that first meet the eye, and a multitrophic level perspective such as is ubiquitous for macroscopic habitats could significantly enhance our understanding of these microscopic worlds. As studies which address structure and function across domains and trophic levels become more common, it will become apparent how interactions within complex microbial jungles such as stream biofilms regulate their response towards environmental change, and how this impacts the ecosystem fluxes that they mediate.

## DATA ACCESSIBILITY

The raw metatranscriptomics sequences are available at the NCBI Short Read Archive under the accession numbers SRA6043551–SRA6043559 (BioProject PRJNA354225, BioSample accession numbers SAMN06043551–SAMN06043559). In addition, the mRNA reads are available via MGRAST (accession numbers 4532565–4532573). Processed data are available via Dryad (https://doi.org/10.5061/dryad.gj3vj).

## AUTHOR CONTRIBUTION

The experiment was designed and planned by M.M.B., K.W. and T.J.B. The microcosm experiment was performed and the samples were processed by M.M.B. and K.W. Metatranscriptomic library preparation and sequencing were performed by C.S. and T.U. The data were analysed by M.M.B. with significant contributions from T.U. The manuscript was written by M.M.B. with contributions from T.J.B., T.U. and C.S.
