# Supplementary material for: Light availability impacts structure and function of phototrophic stream biofilms across domains and trophic levels
Source: Mol Ecol. 2018 Jun 4;27(14):2913–25. doi: 10.1111/mec.14696 (PMC6055792; doi:10.1111/mec.14696)
Supplement: Supplementary file 1 [file MEC-27-2913-s001.docx]

**Supplemental Information for:**

**Light availability impacts structure and function of phototrophic stream biofilms across domains and trophic levels**

Mia M. Bengtsson, Karoline Wagner, Clarissa Schwab, Tim Urich and Tom J. Battin

**Table of Contents:**

| **Table S1** | Page 2 |
| --- | --- |
| **Table S2** | Page 7 |
| **Table S3** | Page 19 |
| **Figure S1** | Page 21 |
| **Figure S2** | Page 22 |
| **Figure S3** | Page 23 |
| **Figure S4** | Page 24 |

Table S1: Dominant and sub-dominant genus-level taxa. Taxa classified as chironomids were excluded from the list due to their stochastic abundances. Relative abundances are expressed as mean percentages of total assigned SSU rRNA reads (excluding chironomids) in each light availability treatment. The taxa are ordered according to abundance.

| **Dominant taxa** |  |  |  |
| --- | --- | --- | --- |
|  | High light | Intermediate light | Low light |
| Achnanthidium | 10.10 | 12.31 | 9.59 |
| Leptolyngbya | 5.15 | 6.59 | 4.67 |
| Encyonema | 2.89 | 1.21 | 0.84 |
| Nitzschia | 2.02 | 1.31 | 1.38 |
| FJ382000 | 1.52 | 0.93 | 0.54 |
| Naviculales (Plastid) | 0.52 | 0.66 | 1.73 |
| Chamaesiphon | 0.60 | 0.55 | 1.24 |
| Gomphonema | 0.77 | 0.43 | 0.61 |
| Pseudendoclonium (Plastid) | 0.65 | 0.77 | 0.35 |
| Environmental samples (Cercomonadidae) | 0.48 | 0.54 | 0.55 |
| unclassified.Rhodophyta (Plastid) | 0.42 | 0.54 | 0.54 |
| Kappamyces | 0.44 | 0.39 | 0.53 |
| Rhizophydium | 0.46 | 0.33 | 0.42 |
| Arachnula | 0.37 | 0.38 | 0.42 |
| Flexibacter | 0.44 | 0.46 | 0.25 |
| Glaeseria | 0.37 | 0.49 | 0.28 |
| Chrysochaete | 0.53 | 0.44 | 0.12 |
| Haliscomenobacter | 0.33 | 0.38 | 0.37 |
| Nuclearia | 0.28 | 0.40 | 0.32 |
| Chytriomyces | 0.43 | 0.27 | 0.16 |
| Chilodonella | 0.27 | 0.25 | 0.20 |
| Opitutus | 0.23 | 0.24 | 0.22 |
| Diatoma | 0.34 | 0.23 | 0.08 |
| Platyreta | 0.22 | 0.19 | 0.23 |
| CU466744 | 0.22 | 0.27 | 0.10 |
| Holosticha | 0.09 | 0.17 | 0.31 |
| Aspidisca | 0.14 | 0.12 | 0.28 |
| Dickieia | 0.17 | 0.18 | 0.17 |
| Pseudodifflugia | 0.22 | 0.14 | 0.15 |
| Colpodidium | 0.25 | 0.22 | 0.04 |
| Sphenomonadales | 0.17 | 0.16 | 0.16 |
| Flavobacterium | 0.13 | 0.15 | 0.21 |
| Trithigmostoma | 0.19 | 0.17 | 0.11 |
| Paraphysomonas | 0.17 | 0.17 | 0.11 |
| Cephalodella | 0.17 | 0.16 | 0.11 |
| Prosthecobacter | 0.10 | 0.16 | 0.17 |
| Luteolibacter | 0.04 | 0.09 | 0.30 |
| Korotnevella | 0.17 | 0.17 | 0.08 |
| Nolandella | 0.15 | 0.15 | 0.12 |
| Cercomonas | 0.14 | 0.16 | 0.11 |
| Echinamoeba | 0.20 | 0.13 | 0.07 |
| Amastigomonas | 0.14 | 0.15 | 0.10 |
| Rickettsia | 0.10 | 0.15 | 0.13 |
| Fistulifera | 0.07 | 0.08 | 0.22 |
| OPB35.soil.group | 0.06 | 0.10 | 0.19 |
| Sandarakinorhabdus | 0.13 | 0.12 | 0.08 |
| EU340220 | 0.08 | 0.13 | 0.10 |
| Neobodo | 0.08 | 0.08 | 0.15 |
| Paracercomonas | 0.13 | 0.14 | 0.03 |
| Prochlorothrix | 0.16 | 0.10 | 0.04 |
|  |  |  |  |
|  |  |  |  |
| **Sub-dominant taxa** |  |  |  |
|  |  |  |  |
| Sphaerotilus | 0.05 | 0.07 | 0.18 |
| Hyalodiscaceae | 0.08 | 0.13 | 0.07 |
| Navicula | 0.06 | 0.07 | 0.15 |
| FJ849203 | 0.05 | 0.09 | 0.13 |
| Armatimonadetes | 0.04 | 0.08 | 0.15 |
| Chattonella (Mitochondrion) | 0.09 | 0.08 | 0.10 |
| Sphaeroeca | 0.11 | 0.09 | 0.06 |
| Haloferula | 0.02 | 0.04 | 0.19 |
| vadinHA49 | 0.04 | 0.09 | 0.11 |
| env.OPS.17 | 0.08 | 0.09 | 0.08 |
| Tychonema | 0.07 | 0.08 | 0.08 |
| Lobulomyces | 0.09 | 0.09 | 0.05 |
| Arcicella | 0.05 | 0.06 | 0.12 |
| Cytophaga | 0.06 | 0.09 | 0.06 |
| Platyamoeba | 0.04 | 0.05 | 0.13 |
| NS9 marine group | 0.04 | 0.06 | 0.12 |
| OPB56 | 0.07 | 0.10 | 0.04 |
| Lembadion | 0.12 | 0.09 | 0.01 |
| Klebsormidium | 0.05 | 0.04 | 0.12 |
| Rhodobacter | 0.06 | 0.05 | 0.09 |
| Microcystis | 0.11 | 0.06 | 0.03 |
| Synedra | 0.10 | 0.06 | 0.04 |
| Candidate division OD1 | 0.04 | 0.04 | 0.12 |
| AJ307936 | 0.04 | 0.08 | 0.07 |
| AY151723 | 0.05 | 0.07 | 0.07 |
| Candidatus Captivus | 0.05 | 0.07 | 0.07 |
| SWB04 | 0.05 | 0.09 | 0.05 |
| HM319234 | 0.04 | 0.09 | 0.06 |
| Gemmata | 0.02 | 0.04 | 0.12 |
| Haliangium | 0.03 | 0.06 | 0.09 |
| Chthoniobacter | 0.02 | 0.04 | 0.12 |
| Nannocystis | 0.05 | 0.07 | 0.06 |
| Sorangium | 0.04 | 0.05 | 0.08 |
| Anoplophrya | 0.03 | 0.08 | 0.07 |
| Zygnemophyceae (Plastid) | 0.07 | 0.05 | 0.06 |
| ML635J.21 | 0.03 | 0.03 | 0.11 |
| GOBB3.C201 | 0.03 | 0.05 | 0.09 |
| Chloroflexi Subdivision 2 | 0.04 | 0.07 | 0.06 |
| Thalassiosirales (Plastid) | 0.02 | 0.04 | 0.11 |
| AB527076 | 0.09 | 0.05 | 0.02 |
| Roseomonas | 0.07 | 0.05 | 0.03 |
| Platyophrya | 0.04 | 0.04 | 0.07 |
| Planomonas | 0.05 | 0.06 | 0.03 |
| Euplotes | 0.07 | 0.06 | 0.01 |
| JG37.AG.15 | 0.04 | 0.05 | 0.06 |
| Goniomonas | 0.08 | 0.05 | 0.02 |
| Massisteria | 0.05 | 0.06 | 0.03 |
| Hymenobacter | 0.05 | 0.06 | 0.03 |
| Xanthomonas | 0.06 | 0.05 | 0.03 |
| Rhodospirillaceae bacterium Dia 1 | 0.07 | 0.05 | 0.00 |

**Table S2:** Taxa which show a differential abundance of rRNA between the highest and lowest light treatments. Positive fold change values indicate significantly higher abundance under high light, while negative values indicate significantly lower abundance under high light (i.e. higher abundance under low light). Testing was performed employing negative binomial GLMs as implemented in the edgeR R package. P-values were corrected for multiple testing using the Benjamini-Hochberg method.

| **Phylum-Kingdom-Class level** | **Genus level** | **Fold change (logFC)** | **P-value (FDR)** |
| --- | --- | --- | --- |
| Acidobacteria |  | -1.751 | <0.001 |
|  | Acidobacteria group 6 (DA023) | -2.073 | <0.001 |
|  | 11-24 | -2.562 | 0.001 |
|  | Candidatus Solibacter | -2.189 | 0.009 |
|  | Bryobacter | -1.372 | 0.022 |
|  | Candidatus Chloracidobacterium | -1.689 | 0.033 |
| Actinobacteria |  | -1.136 | 0.039 |
|  | TM214 | -2.412 | 0.003 |
|  | Iamia | -1.687 | 0.005 |
|  | CL500-29 marine group | -1.254 | 0.009 |
| Alphaproteobacteria |  |  |  |
|  | Nordella | -2.226 | <0.001 |
|  | HM057618 | 5.905 | <0.001 |
|  | Rhodospirillaceae bacterium Dia-1 | 4.316 | 0.001 |
|  | Meganema | -2.402 | 0.001 |
|  | Roseomonas | 1.274 | 0.001 |
|  | Sphingopyxis | -2.690 | 0.001 |
|  | EU861930 | 3.037 | 0.003 |
|  | Hirschia | -1.339 | 0.019 |
|  | Paracoccus | -2.811 | 0.008 |
|  | Bosea | 1.575 | 0.022 |
|  | GOBB3-C201 | -1.173 | 0.023 |
|  | DB1-14 | 1.516 | 0.025 |
|  | Sandarakinorhabdus | 0.962 | 0.025 |
|  | Roseococcus | 1.366 | 0.030 |
|  | Rhodovarius | 1.785 | 0.050 |
| Aquificae |  | -3.822 | 0.001 |
|  | Thermocrinis | -3.095 | 0.035 |
| Armatimonadetes |  | -1.743 | <0.001 |
|  | Armatimonadetes | -1.513 | <0.001 |
| Bacteroidetes |  |  |  |
|  | vadinHA17 | 6.773 | <0.001 |
|  | EU101256 | 5.005 | <0.001 |
|  | Indibacter alkaliphilus | -6.014 | <0.001 |
|  | NS9 marine group | -1.329 | 0.001 |
|  | vadinBC27 wastewater-sludge group | 2.325 | 0.001 |
|  | Flectobacillus | 3.902 | 0.001 |
|  | DUNssu136 | 1.770 | 0.001 |
|  | Prolixibacter | 2.142 | 0.001 |
|  | AB199577 | -2.252 | 0.001 |
|  | RC9 gut group | 2.590 | 0.001 |
|  | Fabibacter | 3.535 | 0.003 |
|  | Microscilla | 2.987 | 0.003 |
|  | Saprospira | 2.562 | 0.004 |
|  | Siphonobacter | 1.713 | 0.038 |
|  | Dysgonomonas | 4.571 | 0.041 |
|  | FJ849114 | -2.166 | 0.008 |
|  | LiUU-11-161 | 1.467 | 0.008 |
|  | Ferruginibacter | -1.210 | 0.009 |
|  | Flexibacter | 1.106 | 0.009 |
|  | Adhaeribacter | 1.803 | 0.009 |
|  | GU451660 | 5.093 | 0.014 |
|  | EF494316 | 2.494 | 0.014 |
|  | Sphingobacterium | 2.031 | 0.015 |
|  | FJ849220 | -1.341 | 0.017 |
|  | EU283531 | 2.640 | 0.017 |
|  | DQ130036 | 3.373 | 0.019 |
|  | Leadbetterella | 1.399 | 0.021 |
|  | CU466744 | 1.508 | 0.033 |
|  | NS11-12 marine group | 1.077 | 0.050 |
|  | DQ917823 | 4.519 | 0.050 |
| Betaproteobacteria |  |  |  |
|  | Limnobacter | -3.899 | <0.001 |
|  | DQ521527 | 2.103 | 0.001 |
|  | MWH-UniP1 aquatic group | -3.694 | 0.001 |
|  | Zoogloea | -5.001 | 0.003 |
|  | Limnohabitans | -2.208 | 0.035 |
|  | CM1G08 | -2.177 | 0.007 |
|  | Sphaerotilus | -1.649 | 0.008 |
|  | Rhizobacter | -1.464 | 0.015 |
|  | beta proteobacterium BP-5 | -4.418 | 0.025 |
| Candidate division BRC1 |  | 1.289 | 0.041 |
|  | Candidate division BRC1 | 1.517 | 0.024 |
| Candidate division OD1 |  | -1.611 | 0.013 |
| Candidate division SPAM |  | -2.187 | 0.027 |
| Chlorobi |  |  |  |
|  | OPB56 | 1.144 | 0.007 |
| Chloroflexi |  | -0.964 | 0.044 |
|  | Chloroflexi Subdivision 8 - TK10 | -3.087 | 0.015 |
| Deltaproteobacteria |  |  |  |
|  | Phaselicystis | -1.631 | 0.001 |
|  | Peredibacter | 1.711 | 0.035 |
|  | Rs-K70 termite group | 4.909 | 0.014 |
|  | HM267229 | 1.231 | 0.027 |
|  | GQ264046 | 4.629 | 0.028 |
|  | Haliangium | -1.176 | 0.032 |
|  | OM27 clade | -0.928 | 0.033 |
| Fibrobacteres |  | -4.986 | 0.001 |
| Gammaproteobacteria |  |  |  |
|  | Nevskia | -3.848 | 0.004 |
|  | Acinetobacter | -2.719 | 0.010 |
|  | Alkanindiges | -1.800 | 0.026 |
|  | Marinicella | -2.726 | 0.027 |
|  | GQ441348 | -2.619 | 0.044 |
| Gemmatimonadetes |  | -0.928 | 0.050 |
|  | Gemmatimonas | -1.066 | 0.047 |
| Lentisphaerae |  | -2.966 | <0.001 |
| Planctomycetes |  | -1.494 | <0.001 |
|  | Gemmata | -2.138 | <0.001 |
|  | Planctomyces | -1.929 | <0.001 |
|  | BD7-11 | -2.703 | 0.004 |
|  | Blastopirellula | -4.741 | 0.017 |
|  | OM190 | -1.882 | 0.007 |
|  | Schlesneria | -2.983 | 0.014 |
|  | CCM11a | -4.479 | 0.025 |
| SHA-109 |  | -2.297 | 0.000 |
|  | SHA-109 | -1.706 | 0.003 |
| Spirochaetes |  | 1.215 | 0.017 |
|  | Leptospira | 1.548 | 0.001 |
| TA18 |  | -2.933 | 0.017 |
| Verrucomicrobia |  | -1.270 | 0.002 |
|  | GQ396806 | -3.950 | <0.001 |
|  | OPB35 soil group | -1.502 | <0.001 |
|  | Chthoniobacter | -2.018 | <0.001 |
|  | Haloferula | -2.719 | <0.001 |
|  | Luteolibacter | -2.600 | <0.001 |
|  | FJ437760 | -5.294 | 0.001 |
|  | FJ437945 | -2.541 | 0.024 |
|  | HM262900 | -4.233 | 0.038 |
|  | AF418948 | -4.212 | 0.041 |
|  | GQ340079 | -4.196 | 0.042 |
| Cyanobacteria |  |  |  |
|  | Pseudanabaena Clusters 4 and 5 | 2.406 | <0.001 |
|  | GQ441281 | -2.866 | <0.001 |
|  | Prochlorothrix | 2.434 | <0.001 |
|  | Calothrix | -3.966 | <0.001 |
|  | GQ397093 | -3.026 | <0.001 |
|  | Chroococcidiopsis Cluster 1 | -3.435 | <0.001 |
|  | AB527076 | 2.150 | <0.001 |
|  | Microcystis | 2.407 | 0.001 |
|  | Synechococcus Cluster 4 | -2.102 | 0.001 |
|  | FR667354 | -5.387 | 0.001 |
|  | EF438248 | -2.339 | 0.001 |
|  | AB518478 | -2.950 | 0.001 |
|  | Lyngbya | 2.085 | 0.004 |
|  | FJ382000 | 1.720 | 0.004 |
|  | FR667251 | 2.878 | 0.005 |
|  | Chamaesiphon | -0.778 | 0.036 |
|  | Stigonema | 4.828 | 0.021 |
|  | AF076158 | 3.293 | 0.027 |
|  | AB183567 | 3.228 | 0.050 |
|  | ML635J-21 | -1.529 | 0.050 |
| Chrysophyceae |  |  |  |
|  | Chrysochaete | 2.438 | <0.001 |
|  | Ochromonas | 3.109 | <0.001 |
|  | Phaeoplaca | 4.333 | 0.001 |
|  | Poterioochromonas | 5.559 | 0.011 |
|  | Hibberdia | 2.470 | 0.007 |
|  | Chrysosphaera | 4.964 | 0.017 |
| Cryptophyta |  | 1.964 | <0.001 |
|  | Goniomonas | 2.349 | 0.001 |
|  | Rhodomonas | 2.300 | 0.036 |
|  | Katablepharis | 1.729 | 0.042 |
| Bacillariophyta (Plastid) |  | -1.319 | 0.001 |
|  | Thalassiosirales (Plastid) | -2.367 | 0.000 |
|  | Naviculales (Plastid) | -1.414 | 0.005 |
|  | Achnanthales (Plastid) | -4.443 | 0.023 |
| Bacillariophyta |  |  |  |
|  | Planothidium | -5.614 | <0.001 |
|  | Diatoma | 2.440 | <0.001 |
|  | Encyonema | 2.061 | <0.001 |
|  | Sellaphora | -1.639 | <0.001 |
|  | Anomoeoneis | 2.818 | 0.001 |
|  | Synedra | 1.719 | 0.001 |
|  | Cymbella | 1.773 | 0.019 |
|  | Navicula | -0.941 | 0.025 |
|  | Cyclophora | -2.614 | 0.035 |
|  | Corethron | 2.858 | 0.037 |
|  | Pleurosigma | -2.146 | 0.046 |
|  | Eolimna | -1.375 | 0.050 |
|  | Stephanodiscus | -2.192 | 0.050 |
|  | Fistulifera | -1.426 | 0.022 |
|  | Asteroplanus | -3.024 | 0.025 |
| PX clade (Plastid) |  | -2.582 | 0.001 |
| Chrysophyceae |  | 1.415 | 0.001 |
| Chlorophyta |  | 1.166 | 0.008 |
|  | Coleochaete | 1.506 | 0.038 |
|  | Amphikrikos | 3.014 | 0.000 |
|  | Klebsormidiophyceae (Plastid) | -2.565 | 0.003 |
|  | Mesotaenium | 2.206 | 0.003 |
|  | AY220083 | 2.808 | 0.017 |
|  | Chaetophora (Chaetophoraceae) | 2.790 | 0.046 |
| Glaucophyta |  |  |  |
|  | Cyanophora | 5.726 | 0.001 |
| Haptophyta |  |  |  |
|  |  |  |  |
|  | Braarudosphaera | -2.302 | 0.037 |
|  |  |  |  |
| Dictyochophyceae |  | 2.014 | 0.037 |
|  | Apedinella | 5.541 | 0.017 |
| Discoba |  |  |  |
|  | Naegleria | -1.886 | 0.019 |
| Rhodophyta (Plastid) |  |  |  |
|  | Palmaria (Plastid) | -3.422 | 0.004 |
|  | Paralemanea (Plastid) | -3.419 | 0.008 |
| Xanthophyceae (Plastid) |  |  |  |
|  | Xanthophyceae (Plastid) | -2.656 | 0.006 |
| Slopalinida |  | 3.809 | <0.001 |
| Centroheliozoa |  | 1.477 | 0.001 |
|  | Heterophrys | 1.767 | 0.001 |
|  | Pterocystis | 1.660 | 0.007 |
|  | Chlamydaster | 2.269 | 0.024 |
| Gastrotricha |  | -2.167 | 0.016 |
| Alveolata |  |  |  |
|  | Dileptus | 4.054 | <0.001 |
|  | Lembadion | 3.234 | <0.001 |
|  | Prorodon | 7.148 | <0.001 |
|  | Glenodinium | 5.868 | 0.001 |
|  | Pseudouroleptus | 4.094 | 0.001 |
|  | Dysteria | 2.070 | 0.001 |
|  | Cyrtolophosis | 2.241 | 0.001 |
|  | Vorticella | 2.548 | 0.003 |
|  | Euplotes | 2.648 | 0.003 |
|  | Paramecium | 2.756 | 0.003 |
|  | Lacrymaria | 1.494 | 0.004 |
|  | Coleps | 2.937 | 0.004 |
|  | Bryometopus | 1.715 | 0.005 |
|  | Levicoleps | 3.218 | 0.011 |
|  | Holosticha | -1.555 | 0.012 |
|  | Colpodidium | 2.890 | 0.017 |
|  | Oxytricha | 4.784 | 0.023 |
|  | Thecadinium | 4.598 | 0.035 |
|  | Chlamydodon | 3.149 | 0.038 |
|  | Enchelyodon | 4.850 | 0.044 |
|  | Hemigastrostyla | 4.889 | 0.019 |
|  | Bardeliella | 2.407 | 0.025 |
| Fungi |  |  |  |
|  | Karlingiomyces | 3.311 | 0.004 |
|  | Chytriomyces | 1.719 | 0.005 |
|  | Serpula (Serpulaceae) | 4.854 | 0.036 |
|  | Pilobolus | -4.218 | 0.041 |
|  | Chytridium | 1.797 | 0.041 |
|  | Cladochytrium | -2.690 | 0.047 |
|  | Dipodascus | -2.763 | 0.022 |
|  | Spizellomyces | -2.563 | 0.027 |
|  | Synchytrium | 4.494 | 0.045 |
|  | Lobulomyces | 1.306 | 0.049 |
| Rhizaria |  |  |  |
|  | Paracercomonas | 2.205 | <0.001 |
|  | Heteromita | 3.440 | <0.001 |
|  | unclassified Cercomonadida | -5.638 | <0.001 |
|  | Hedriocystis | 1.192 | 0.037 |
| Rotifera |  |  |  |
|  | Mniobia | 2.256 | 0.024 |
| Amoebozoa |  |  |  |
|  | Echinamoeba | 1.686 | <0.001 |
|  | Platyamoeba | -1.349 | 0.015 |
|  | Korotnevella | 1.279 | 0.048 |
| Choanomonada |  |  |  |
|  | Sphaeroeca | 1.097 | 0.010 |
|  | Desmarella | 1.872 | 0.019 |
| Ichtyosporea |  |  |  |
|  | Anurofeca | 3.460 | 0.003 |
| Insecta |  |  |  |
|  | Oxyethira | -6.070 | 0.003 |
| Opalinata |  |  |  |
|  | Karotomorpha | 4.004 | <0.001 |
| Bicosoecida |  |  |  |
|  | Adriamonas | 1.718 | 0.009 |

**Table S3:** Differentially expressed gene categories (Subsystems) over the whole dataset (overall) and assigned to major taxonomical groups of biofilm phototrophs and heterotrophic bacteria. Values indicate the fold-change in expression between the extreme light treatments (highest light vs. lowest light). Positive values signify increased expression under high light, while negative values signify decreased expression under high light (i.e. increased expression under low light). Only significant values are displayed (p < 0.05 after correction for multiple testing using the Benjamini-Hochberg method). Testing was performed employing negative binomial GLMs as implemented in the edgeR R package.

| **Subsystems**  **level 1** | **Subsystems**  **level 2** | **Overall** | **Bacillariophyta (including plastid)** | **Cyanobacteria** | **Chlorophyta (including plastid)** | **Beta-proteobacteria** | **Alpha-proteobacteria** | **Gamma-proteobacteria** | **Heterotrophic bacteria (Bacteria-Cyanobacteria)** |
| --- | --- | --- | --- | --- | --- | --- | --- | --- | --- |
| Photosynthesis | |  |  | -0.716 |  |  |  |  |  |
|  | Electron transport and phosphorylation |  | -0.533 | -0.810 |  |  |  |  |  |
| Protein metabolism | |  |  |  |  |  |  |  |  |
|  | Protein folding | 0.960 |  | 1.110 |  |  |  |  |  |
|  | Protein export |  | -4.443 |  |  |  |  |  |  |
|  | Protein degradation |  | 1.364 |  |  |  |  |  |  |
| Respiration |  |  | -0.877 | -0.538 |  |  |  |  |  |
|  | - |  |  |  | 5.264 |  |  |  |  |
| Phosphorous metabolism | | 1.559 |  | 2.669 |  | 1.929 | 2.062 |  | 1.614 |
|  | Phosphate metabolism | 2.317 |  | 3.582 |  | 2.838 |  |  | 2.753 |
| Iron acquisition and metabolism | | 0.538 |  |  |  |  |  |  |  |
| Phages, Prophages, Transposable elements, Plasmids | | 0.700 |  |  |  |  |  | 2.209 | 0.879 |
|  | Pathogenicity islands | 1.112 |  |  |  |  |  |  |  |
| Fatty Acids, Lipids, and Isoprenoids | |  |  |  |  |  |  |  |  |
|  | Isoprenoids |  |  |  |  |  |  | -3.685 |  |
| Virulence, Disease and Defense | |  |  |  |  |  |  |  | -0.753 |
| Clustering-based subsystems | |  |  |  |  |  |  |  |  |
|  | Hypothetical lipase related to Phosphatidate metabolism | -3.349 |  |  |  |  |  |  |  |
| Cofactors, Vitamins, Prosthetic Groups, Pigments | |  |  |  |  |  |  |  |  |
|  | Coenzyme A | 1.382 |  |  |  |  |  |  |  |
| Metabolism of Aromatic Compounds | |  |  |  |  |  |  |  |  |
|  | Metabolism of central aromatic intermediates | -1.615 |  |  |  |  |  |  |  |

Figure S1: The relative abundances of the most abundant diatom genera (Bacillariophyta) in

relation to light intensity.

Figure S2: The relative abundances of the most abundant cyanobacterial genera in relation

to light intensity.

Figure S3: The relative abundances of SSU rRNA from putatively phototrophic taxa (green

shades), putatively heterotrophic bacteria (blue shades) and putatively heterotrophic

eukaryotes, a.k.a. consumers (red shades) in relation to light intensity.

Figure S4: The relative abundances of the most abundant Alveolata genera (all ciliates) in

relation to light intensity.
